# Effect of glutaraldehyde on thermal and mechanical properties of starch and polyvinyl alcohol blends

**DOI:** 10.1080/15685551.2019.1678222

**Published:** 2019-10-23

**Authors:** Ravindra V. Gadhave, Prakash A. Mahanwar, Pradeep T. Gadekar

**Affiliations:** Department of Polymer and Surface Engineering, Institute of Chemical Technology, Mumbai, India

**Keywords:** Starch, polyvinyl alcohol, cross-linking, glutaraldehyde, mechanical testing

## Abstract

The aim of this study is to analyze the various compositions of polyvinyl alcohol (PVA) and starch (S) blends. The blends have been cross-linked with glutaraldehyde to enhance its properties. The hydroxyl groups of PVA and starch react with glutaraldehyde via formation of acetal bonds hence crosslinking could take place. The cross-linking of glutaraldehyde is observed with the help of various analytical methods such as differential scanning calorimetry (DSC) and Fourier-transform infrared spectroscopy (FTIR). The presence of two highly reactive alpha protons makes glutaraldehyde more reactive and acidic in nature. The higher reactivity of glutaraldehyde, at higher dosages leads to reduction in H-bonding of PVA and starch. The cross-linked blends showed better thermal and mechanical properties. Viscosity, tensile strength, pencil hardness, and ultimate stress were evaluated to estimate the changes due to cross-linking. It was observed that the mechanical properties are directly proportional to the amount of starch as the starch hydroxyl groups are easily accessible for the cross-linking reaction. The cross-linked blend showed better cohesion between its chains, thereby increasing the glass transition temperature. It was reflected in the subsequent increase in tensile strength properties.

## 1. Introduction

Starch is a relatively inexpensive and renewable product that can be obtained from multiple plant sources and that has been extensively used as a wood adhesive [1,2]. However, its bonding capacity is not strong enough to glue wood [3–7]. A few studies have been conducted on the potential of utilizing starch as wood adhesive. Recent studies have focused on formaldehyde-free wood adhesives which are obtained through the reaction between a cross-linker and a blend of starch with other polymers, such as polyvinyl alcohol [8], tannin [9–11] and isocyanates [12].

Polyvinyl alcohol (PVA) is a water-soluble biodegradable polymer. It possesses a high crystalline structure [13]. Physical and chemical properties of PVA depend on the synthesis condition and degree of hydrolysis of the polymer [14–18]. To improve the properties, there are a wide variety of crosslinking agents for PVA, such as maleic acid, formaldehyde, and glutaraldehyde [19].

PVA cross linked with glutaraldehyde is one of the most commonly used techniques. It is well known that hydroxyl groups from PVA react with glutaraldehyde via formation of acetal bonds [20,21]. Glutaraldehyde is a multifunctional reagent that cross-links starch by reacting with the hydroxyl groups of starch thereby introducing intermolecular bridges between the polysaccharide chains.

## 2. Experimental

### 2.1. Materials

PVA (containing 87–89% degree of hydrolysis) was obtained from Kuraray Cooperative Limited, India. Maize starch containing 25–30% of amylose content was obtained from Sanstar Ltd. Glutaraldehyde (25% solution) was purchased from Sigma-Aldrich. Maize starch was dried to remove the moisture.

### 2.2. Preparation method

Maize starch and PVA were mixed in water and poured in a four-necked flask equipped with stirrer and reflux condenser. The reaction flask was heated to 60°C while mixing the sample at 175 rpm. The cross-linker was later added as per the amount given in Table 1. Subsequently, the temperature was raised to 92-95°C and maintained for 2.5 h. After completion of the cross-linking, the solution was brought to room temperature for further analysis.

**Table 1.** Preparation of various adhesive compositions with glutaraldehyde.

	Blends	PVA	Starch	Glutaraldehyde	Water
Formulation 1	PVA/S-07	25	5	0	70
	PVA/S-GLU.01	25	5	0.5	70
	PVA/S-GLU.02	25	5	1.0	70
	PVA/S-GLU.03	25	5	1.5	70
Formulation 2	PVA/S-08	20	10	0	70
	PVA/S-GLU.04	20	10	0.5	70
	PVA/S-GLU.05	20	10	1.0	70
	PVA/S-GLU.06	20	10	1.5	70

### 2.3. Casting of films

A bar applicator was used for casting films of 1000 µm. The films were then kept at room temperature for 24 h to facilitate the curing reaction.

Formulation 1

For formulation 1, the PVA/S blend was kept at a constant ratio of 25/5 with composition of glutaraldehyde varied from 0 to 1.5 (Table 1).

Formulation 2

For formulation 2, the PVA/S blend was kept at a constant ratio of 20/10 with composition of glutaraldehyde varied from 0 to 1.5 (Table 1).

## 3. Characterization and testing

### 3.1. Viscosity

A *Brookfield DV1 Viscometer* was used for calculating the viscosities of the formulations 1 and 2. All readings were taken at 30°C.

### 3.2. Fourier transform infrared spectroscopy (FTIR)

Infrared spectra of crosslinked PVA and starch blends were measured on a PerkinElmer FTIR spectrum 100 instrument. Thin films were made from crosslinked blends prior to analysis.

### 3.3. Differential scanning calorimetry (DSC)

A Perkin Elmer instrument Q100 DSC has been used for estimating the T_g_.

### 3.4. Dynamic mechanical analysis (DMA)

DMA was performed using DMA Q800. A thin film of 200 µm was first prepared by applying it on a PTFE sheet. The film was kept for curing at room temperature for 24 h. After curing, the film was peeled off from the surface and kept in the DMA sample holder. It is a technique used to study and characterize materials. It is the most useful technique for studying the viscoelastic behavior of polymers. A sinusoidal stress was applied and the strain in the material was measured in terms of complex modulus. The temperature of the sample or the frequency of the stress is often varied leading to variations in the modulus. This approach can be used to locate the glass transition temperature of the material as well as to identify transitions corresponding to crosslinking between the molecules.

### 3.5. Pencil hardness test

The testing method of ASTM D 3363 was employed to calculate pencil hardness.

### 3.6. Ultimate stress of films

Tinus Olsen 5ST instrument was used for determining the ultimate stress of the films.

### 3.7. Tensile shear strength

Tinus Olsen H25KTinstrument was used for calculating the tensile shear strength. Two pieces of steamed beech wood were taken for determining the tensile shear strength. The adhesive was applied on one end (2.5 cm × 2.5 cm) of the two pieces and were held together for 2 and 24 h at room temperature. The cured wood samples were then tested using a controlled speed of 10mm/min to obtain the tensile shear strength values.

## 4. Results and discussion

**Figure 1.**
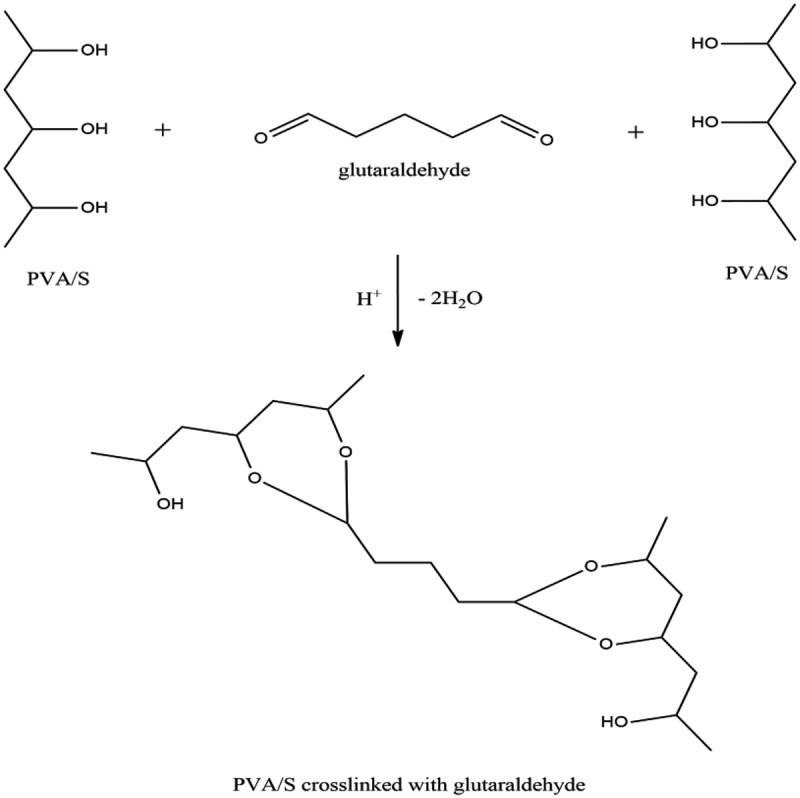
Crosslinking mechanism of PVA/S with glutaraldehyde.

The presence of hydroxyl groups in the structure of PVA and S make the reaction with glutaraldehyde feasible. At 90°C, glutaraldehyde reacts with the hydroxyl groups of PVA and starch forming a cross linked network. The medium required for this reaction to occur is acidic, which is inherent in the solution. The cross-linking mechanism of PVA/S with glutaraldehyde is given below in Figure 1. The results obtained by various tests are given in Table 2 (Formulation 1) and Table 3 (Formulation 2).

**Table 2.** Results obtained by carrying out various tests for Formulation 1.

	Viscosity (poise)	Ultimate stress	T_g_ (°C)	Pencil hardness	Tensile strength (kg/sq.in.)	
					4 h	24 h
PVA/S-07	46	7.6	65	H	7	11.2
PVA/S-GLU.01	53	6.7	68	7H	8.3	12.2
PVA/S-GLU.02	65	10.8	71	8H	8.5	12.6
PVA/S-GLU.03	70	16.2	73	8B	9.9	12.8

**Table 3.** Results obtained by carrying out various tests for Formulation 2.

	Viscosity (poise)	Ultimate stress	T_g_ (°C)	Pencil hardness	Tensile strength (kg/sq.in.)	
					4 h	24 h
PVA/S-08	88	7.9	69	2H	7.2	9.3
PVA/S-GLU.04	120	11.3	71	7H	7.9	11.3
PVA/S-GLU.05	135	12.6	73	8H	8.6	10.9
PVA/S-GLU.06	147	13.2	74	9H	9.4	12.6

### 4.1. Viscosity

**Figure 2.**
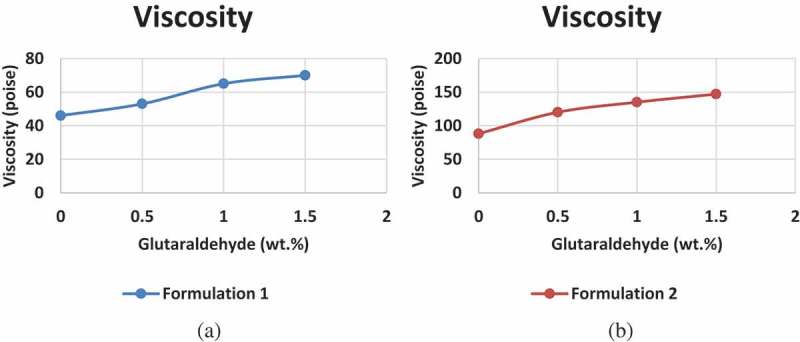
Viscosity at increasing concentration of glutaraldehyde given by (a) Formulation 1and (b) Formulation 2.

The glutaraldehyde acts as a cross-linker for PVA/S and this has led to subsequent viscosity change as shown in Figure 2.

The effect of cross-linking between the hydroxyl groups of PVA/S is evident in Figure 2. The glutaraldehyde has reacted with the hydroxyl groups and thus forming a cross-link. This cross-linking increases the chain length of the PVA and S, thus the increase in viscosity. Since the longer chain length increases the chances of entanglement, it has led to an increase in viscosity as evident in Figure 2. While comparing the formulation 1 and formulation 2 values, we get a much greater viscosity in formulation 2. This is due to the presence of hydrolyzed starch which has contributed to increase in viscosity.

### 4.2. Fourier transform infrared spectroscopy (FTIR)

**Figure 3.**
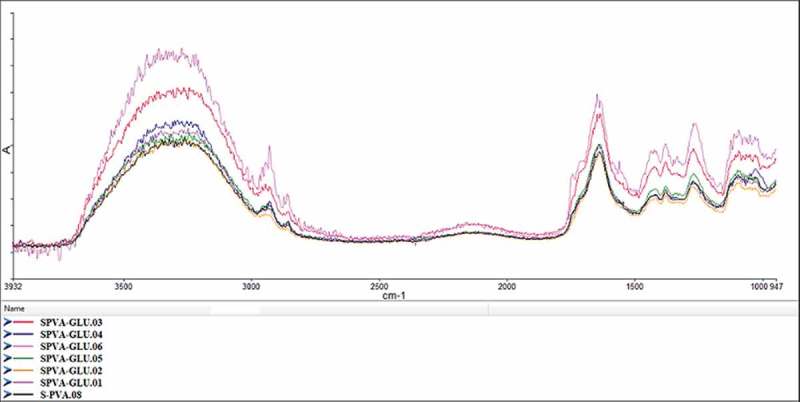
FTIR curves of PVA/S crosslinked with glutaraldehyde.

As the concentration of cross-linker is increased, there is a movement of curve towards the lower wave number. This is observed as the cross-linker reduces the distance between the chains by bridging between them, this bridging leads to lower H-bonding in the adhesive. The presence of two highly reactive alpha protons makes glutaraldehyde more reactive and acidic in nature. The higher reactivity of glutaraldehyde especially at higher dosages leads to reduction in H-bonding of PVA chains. The effect of decreased H-bonding can be seen in Figure 3 as the curve shifts towards lower wave number. Additionally, the increase in starch content also contributes to decrease in H-bonding (Figure 3).

### 4.3. Differential scanning calorimetry (DSC)

**Figure 4.**
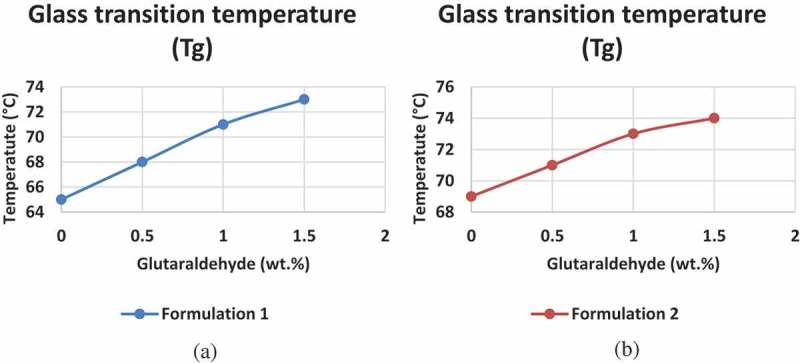
Glass transition temperature (T_g_) at increasing concentration of glutaraldehyde given by (a) Formulation 1 and (b) Formulation 2.

The glutaraldehyde acts as a cross-linker for PVA/S which has led to subsequent glass transition temperature change as shown in Figure 4.

The effect of cross-linking between the hydroxyl groups of PVA/S is evident in Figure 4. The glutaraldehyde has reacted with the hydroxyl groups and thus forming a cross-link. This cross-link increases the chain length of the PVA and S, thus the increase in glass transition temperature. As evident from the FTIR curves, there is a decrease in H-bonding of the adhesive with subsequent increase in the amount of cross-linker. The similar trend is observed for Tg, there is an increase in Tg with increase in the concentration of cross-linker. In comparing formulation 1 and formulation 2, there is slightly higher Tg for formulation 2 due to hydrolysis of starch.

### 4.4. Dynamic mechanical analysis

**Figure 5.**
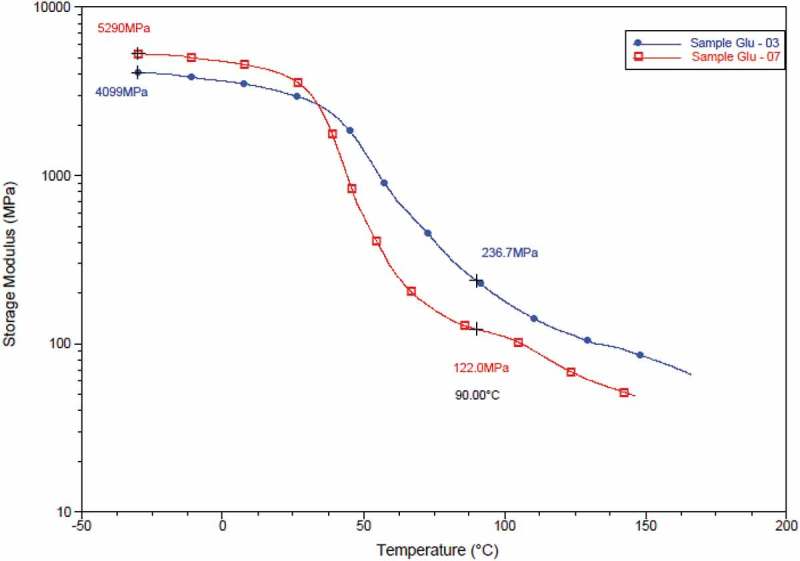
Overlaid thermogram of storage modulus (E’) of samples.

The two samples showed major transition corresponding to polyvinyl alcohol at 90°C. The storage modulus curve shows the highest value for PVA/S-03 throughout the glassy and rubbery region. This confirms the cross-linking effect on the hydroxyl groups of starch and PVA, which has led to increase in the storage modulus. Meanwhile, comparing PVA/S-03 and PVA/S-07, it can be seen that initially at glassy region the PVA/S-07 curve has less modulus (than PVA/S-03) and following transition temperature it has higher modulus (than PVA/S-07) (Figure 5).

The crosslinking has been shown by tan delta curve where there is an evident shift in the peak of the curve towards the higher temperature (from 70.61°C to 82.52°C).

**Figure 6.**
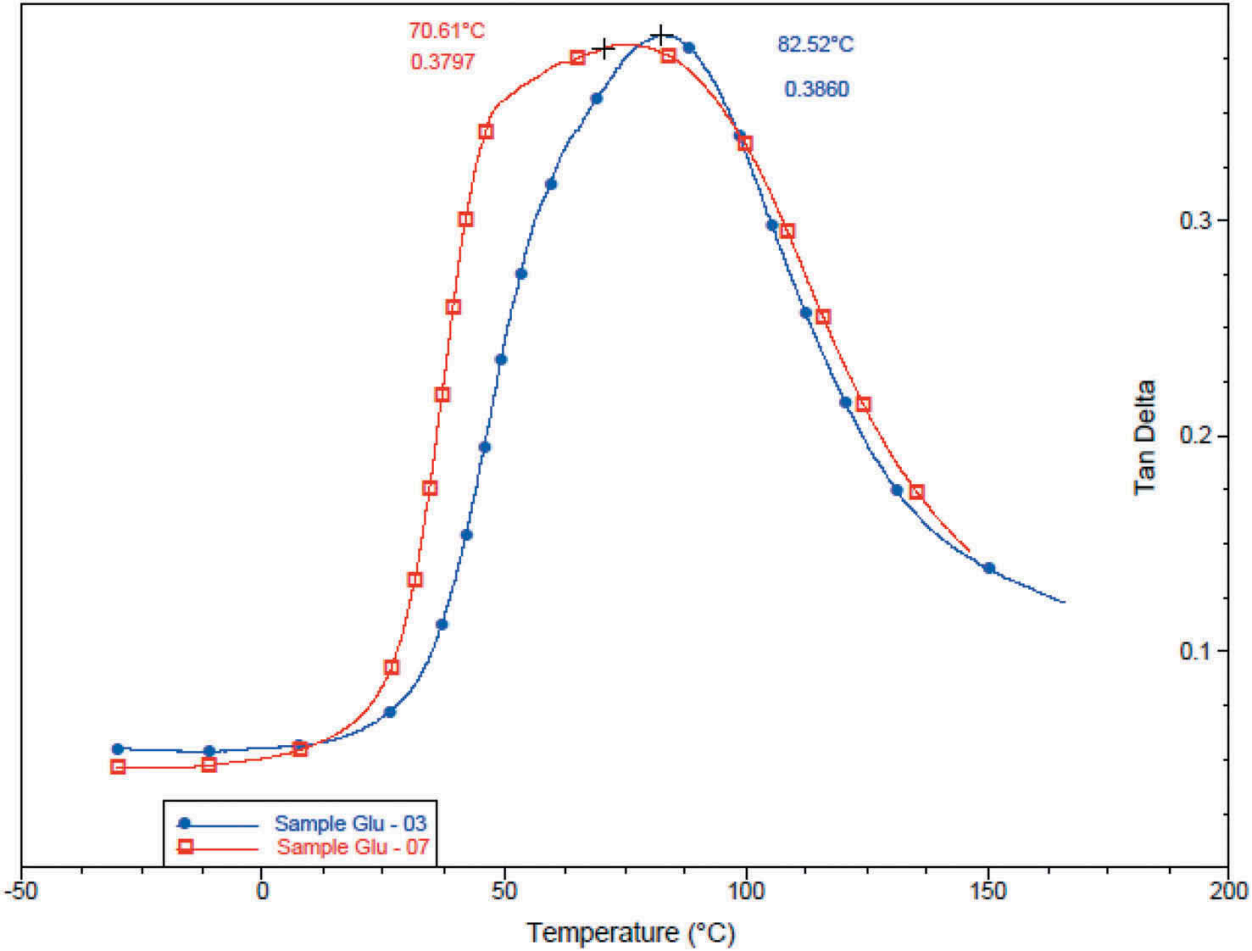
Overlaid thermogram of tan delta of samples.

This shift in peak is a representation of the transition temperature change which has caused due to the cross-linking of PVA and starch blends with glutaraldehyde (Figure 6).

### 4.5. Pencil hardness of film

Pencil hardness is a property dependent on the flexibility of the polymeric chains. The flexible chains of PVA are replaced partially by starch (which contains large six membered rings). The presence of rings in starch has contributed to increase in the hardness of formulation 2. Better cohesion in blends has also contributed to increase in the hardness (as seen in Table 4). Both the formulations show a large difference in hardness. The cross-linking has clearly enhanced the hardness of the blends.

**Table 4.** Effect of cross-linking by glutaraldehyde on pencil hardness of PVA/S blend.

Formulation 1		Formulation 2	
Blends	Pencil hardness	Blends	Pencil hardness
PVA/S-07	H	PVA/S-08	2H
PVA/S-GLU.01	7H	PVA/S-GLU.04	7H
PVA/S-GLU.02	8H	PVA/S-GLU.05	8H
PVA/S-GLU.03	8H	PVA/S-GLU.06	9H

### 4.6. Tensile shear strength

**Figure 7.**
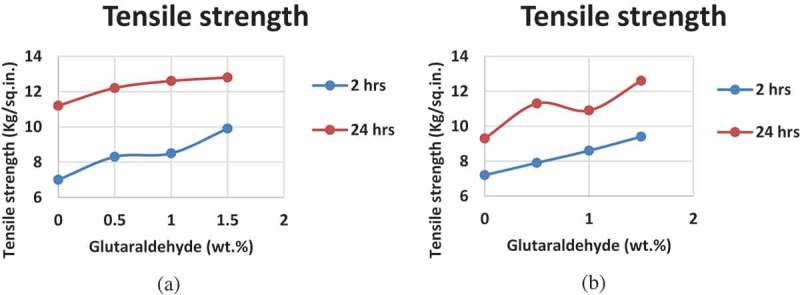
Tensile shear strength with increasing concentration of glutaraldehyde after 2 h and 24 h of (a) Formulation 1 and (b) Formulation 2.

The glutaraldehyde acts as a cross-linker for PVA/S which has led to subsequent tensile strength change as shown in Figure 7.

The tensile strength shows a similar trend as that of viscosity, glass transition temperature and pencil hardness of the blends. As there is a decrease in free volume between the chains, there is an increase in number of hydroxyl groups at the surface and as the hydroxyl groups become abundant on the surface, there is an increase in tensile strength. Since the decrease in the free volume is proportional to the amount of cross linker added, the tensile strength is increased subsequently (Figure 7(a,b)). The cohesion between chains has also increased which further reduces the chances of tensile failure.

### 4.7. Ultimate stress of films

The cross-linking of glutaraldehyde has decreased the mobility of the polymer chains. This has led to increase in stress-bearing capacity of the chains thereby increasing the stress required to break the blends. While comparing the formulation 1 and formulation 2, the presence of more number of six membered rings of starch in formulation 2 helped in increasing its stress-bearing capacity as seen in Table 5.

**Table 5.** Effect of cross-linking by glutaraldehyde on ultimate stress of PVA/starch composite films.

Formulation 1		Formulation 2	
Blends	Ultimate stress	Blends	Ultimate stress
PVA/S-07	7.6	PVA/S-08	7.9
PVA/S-GLU.01	6.7	PVA/S-GLU.04	11.3
PVA/S-GLU.02	10.8	PVA/S-GLU.05	12.6
PVA/S-GLU.03	16.2	PVA/S-GLU.06	13.2

## 5. Conclusions

The cross-linking of PVA/S blends has efficiently increased thermal and mechanical properties. There is an increase in cohesion and decrease in H-bonding which has contributed majorly to increase in viscosity. Mechanical properties such as tensile strength, ultimate stress, and pencil hardness have also shown an enhancing effect due to cross-linking. There is a shift in the glass transition temperature and increase in the area of tan delta curve which support the cross-linking mechanism. The FTIR proves the decrease in H-bonding due to cross-linking by glutaraldehyde. Consequently, the cross-linking has contributed to increase in thermal and mechanical properties. The presence of more starch content is found to increase the cross-linking due to easy accessibility for cross-linker to attack on hydroxyl groups.
